# The Sociology of Suicide After COVID-19: Assessment of the Spanish Case

**DOI:** 10.3390/bs15050606

**Published:** 2025-05-01

**Authors:** Enrique Fernández-Vilas, Juan R. Coca, Juan José Labora González, Marcos Iglesias Carrera

**Affiliations:** 1 Department of Sociology and Social Work, University of Valladolid, José Tudela 12D, 42004 Soria, Spain; juanr.coca@uva.es; 2 Department of Political Science and Sociology, University of Santiago de Compostela, Ángel Jorge Echeverri, 15782 Santiago de Compostela, Spain; juan.labora@usc.es; 3 School of Labor Relations, University of Salamanca, San Torcuato 43, 49014 Zamora, Spain; marcosiglesias@usal.es

**Keywords:** suicide, COVID-19, mental health, suicide prevention, gender differences, socioeconomic inequalities, post-pandemic mental health, public health

## Abstract

The phenomenon of suicide has become a significant global concern, claiming over 800,000 lives annually and resulting in millions of suicide attempts worldwide. In the aftermath of the COVID-19 pandemic, these troubling statistics have worsened, with notable increases in suicidal behavior, especially among vulnerable populations such as the youth, the elderly, and those in socioeconomically disadvantaged groups. This paper aims to explore the impact of the COVID-19 pandemic on suicide rates in Spain, using a theoretical ex post facto analysis. Spain has witnessed an alarming rise in suicide rates, particularly among young people, and a disturbing trend of increased suicidal ideation and self-harm behaviors. While some studies report no significant change in suicide rates during the pandemic, others point to the exacerbating effects of social isolation, economic instability, and public health measures. This study provides an in-depth examination of the psychosocial consequences of the pandemic on mental health in Spain, emphasizing the urgency of the need to address pre-existing inequalities and implement effective suicide prevention measures. Furthermore, it highlights the importance of gender-sensitive strategies and the need for systemic reforms to ensure better mental healthcare access for all segments of society. To achieve this goal, this paper uses a narrative literature review combined with a theoretical ex post facto analysis to assess the influence of the COVID-19 pandemic on suicide patterns in Spain.

## 1. Introduction

The social phenomenon of suicide claims the lives of more than 800,000 individuals globally each year. In addition, it has been estimated that around 16 million suicide attempts are estimated to occur worldwide each year (Clua-García et al., 2021). That is, more than 2000 people die by suicide and almost 45,000 attempt suicide every day. Adult males have been found to be at higher risk of suicide, while young women are more likely to attempt suicide. Nearly one-third of all suicide attempts worldwide are made by young people, making it the second leading cause of death among 15–29 year olds and 15–19-year-old females (Mental Health and Substance Use, 2021).

The most common ways of committing suicide include the use of pesticides, hanging, or firearms, although jumping from high places and overdose are also common (Mental Health and Substance Use, 2021). However, there are significant differences in the choice of method between different countries and genders, with 71% of women opting for poisoning compared to 50% of men (Clua-García et al., 2021). The lethality of these methods varies considerably by region.

In the aftermath of the COVID-19 pandemic, suicide rates and mental health disorders have shown varying trends globally. While some studies found no significant changes in suicide rates during the pandemic (Pathirathna et al., 2022), others reported an increase in suicidal ideation and suicide attempts (Schnitzer et al., 2023). Prolonged social isolation and economic uncertainty have contributed to the deterioration of human well-being and have promoted this phenomenon (Sher, 2020). One of the most affected age groups was children and young people, whose members experienced disruptions to their education and social life. Also, the older age group of the elderly suffered a higher degree of isolation and lost more people they were close to due to death (Yan et al., 2023).

On the other hand, the pandemic led to an increase in gender inequality within suicide rates, with higher rates of suicidal ideation and suicide attempts seen in young women, while men continued to have higher rates of completed suicide due to the use of more lethal methods (Da Cunha Varella et al., 2024).

In Spain, recent studies have shown an increase in the incidence of disorders such as anxiety, depression, and post-traumatic stress disorder during periods of confinement and social restrictions (González-Sanguino et al., 2020). This deterioration in the mental health of the Spanish population has been reflected in an increase in suicide rates. According to data from the Instituto Nacional de Estadística [Spanish Institute of Statistics], 3941 suicides were recorded in 2020 (Instituto Nacional de Estadística, 2021), which represents an increase from the 3671 cases reported in 2019 (Instituto Nacional de Estadística, 2020). This increase is particularly worrying in certain demographic groups. For example, an increase in suicide rates among young people and adolescents during the pandemic has been observed. In 2020, 14 children under the age of 15 died by suicide in Spain, doubling the previous year’s figure (Paricio del Castillo et al., 2024). In addition, the pandemic has exacerbated pre-existing social inequalities, whose impact on groups in situations of socioeconomic vulnerability is clear, contributing to the increase in suicidal behavior (De la Torre Luque et al., 2024).

The aim of this paper is to study the phenomenon of suicide in Spain by means of a theoretical ex post facto analysis. This analysis refers to the examination of suicide trends after the occurrence of the COVID-19 pandemic, without manipulating the variables and instead interpreting the observed consequences. This method is particularly useful in sociology when retrospective interpretation is required.

## 2. Background: COVID-19 and the “New Normal”

In late 2019, several cases of an atypical form of pneumonia in the Wuhan region (China) put health authorities on alert, with the academic community initially referring to these cases as 2019-nCov (2019-novel coronavirus) (Huang et al., 2020). Shortly afterward, and given its taxonomy, the International Committee on Taxonomy of Viruses (ICTV) labelled the causative agent of this disease Sars-CoV-2 (Coronaviridae Study Group of the International Committee on Taxonomy of Viruses, 2020).

More than four years after the onset of the pandemic, over 650 million people have been infected and more than 6.8 million have died from the infection, as reflected in data from Johns Hopkins University (2023), which created an interactive map to monitor real-time disease data.

In addition to the physical deterioration caused by the disease, the distancing and confinement measures implemented led to major stress for the entire population (Manchia et al., 2022). Therefore, it is worth highlighting the relevance of the social study of the psyche in at-risk societies. Great concern exists regarding mental health, at least in relation to the phenomenon of suicide in the Western world. Since the COVID-19 pandemic, with increasing diagnoses of anxiety and depression across the world, concern for the most vulnerable populations, such as children, the elderly, low-income individuals, and those with pre-existing mental health conditions, has been evident in the literature. These groups have experienced heightened levels of stress due to factors like isolation, economic instability, and disrupted healthcare access. Research highlights the need for targeted interventions and support systems to address the unique challenges faced by these populations in coping with the long-term psychological impacts of the pandemic (Holmes et al., 2020; Pappa et al., 2020). Evidence indicates that individuals from lower social classes may have limited access to primary and secondary mental healthcare, leading to a higher likelihood of them seeking help through crisis services. Additionally, patients in lower social classes may not receive the full benefits of mental health interventions, which may be less effective for them (Barnett et al., 2023).

During the months of COVID-19’s greatest impact, there was an increase in the symptoms of other disorders, which have also continued until the present, such as eating disorders (Ji et al., 2024), attention deficit disorder and hyperactivity (Lopes et al., 2022), the appearance of new symptoms and diagnoses after overcoming COVID-19 (Fair Health, 2022), and issues such as insomnia and an ongoing fear of death, among others. But of greatest concern is the rising suicide rates seen since the pandemic. These rates were already high in many countries such as Spain, where the number one cause of unnatural death is now suicide. During 2019, 309 individuals aged 15 to 29 died by suicide in Spain. This same year, 307 people from this same age range died in traffic accidents, making suicide the leading cause of death among this age group.

In 2021, a suicide occurred every two and a half hours in Spain, leading to a total of 4003 suicides during the year (Instituto Nacional de Estadística, 2023). In 2022, suicide remained the leading cause of unnatural death in Spain (Ministerio de Sanidad, 2024). A total of 4097 people died by suicide in Spain during this year, an increase of 2.3% from 2021. Furthermore, the number of suicides increased for individuals under the age of 20 in particular (84 compared to 75 in 2021). Therefore, we can conclude that between 2018 and 2022, there has been an increase in suicide rates in Spain (Instituto Nacional de Estadística, 2023).

Infectious disease outbreaks have been associated with the appearance of mental health disorders in healthcare workers (medical and nursing staff in healthcare systems, social workers in elderly care centers, etc.), who are exposed to great stress given the nature of their work (Aymerich et al., 2022). These workers have faced emergency situations, sick patients, and the possibility of contracting illnesses. This has most likely had a major impact on their mental health, since it has been shown that these workers are at greater risk of developing mental disorders such as depression, generalized anxiety, and post-traumatic stress disorder (Nicolini, 2020). It has also been shown that this is more common in women than in men. Specifically, care and healthcare workers may have developed additional problems during the pandemic, as individuals experiencing significant health-related anxiety are prone to mistakenly perceiving benign physical symptoms as signs of infection (Pearman et al., 2020). This misinterpretation heightens their anxiety, impacts their capacity for logical decision-making, and alters their behavior. This anxiety about their health may simultaneously lead to maladaptive behaviors such as excessive handwashing, social withdrawal, and shopping anxiety (Asmundson & Taylor, 2020).

The uncertainty created by the pandemic has also affected other sectors and social groups, making them particularly vulnerable, such as staff at nursing homes, day centers, survivors, families, and adolescents. This vulnerability includes anxiety and stress associated with the risk of infection, the death of loved ones, uncertainty, social isolation measures, the physical and emotional fatigue of health workers, massive job losses, financial insecurity, poverty, and misinformation and information overload. Contrary to what was suggested during some periods of the pandemic, the literature has shown that the impacts of COVID-19 are correlated with belonging to certain social classes (Bajos et al., 2021; Holst et al., 2021; Lee & Singh, 2021; Soria & Horgos, 2020).

Studying the sociology of health has allowed people to gather evidence on the social processes to be considered in relation to all diseases, as various studies have mentioned (Cockerham, 2021, 2022; Nettleton, 2021). From this perspective, at-risk societies (Beck, 2009) have been subject to a growing concern for their health, mental health, and safety, as reflected in a diverse range of works (e.g., Nettleton, 2021, among others).

The COVID-19 pandemic has underscored and exacerbated pre-existing inequalities across the world, disproportionately affecting various segments of the population in terms of economics, healthcare access, gender, and education (Sohn et al., 2022; Wu & Qian, 2022). Within the Spanish context, these pandemic-amplified inequalities can be linked to an increased vulnerability among certain groups to mental health issues, including the risk of suicide.

The psychosocial impact of the pandemic, characterized by social isolation, job loss, economic uncertainty, and health-related stress, has contributed to a rise in anxiety, depression, and other mental disorders, which are known risk factors for suicide. In Spain, as in many parts of the world, there has been a growing concern for the population’s mental health during and after the pandemic, reflecting the importance of addressing these structural inequalities and their effects on the populace (Husain et al., 2021; Vadivel et al., 2021).

Suicide, as an extreme indicator of psychological distress, can be understood within this context as a manifestation of inadequate support for some social groups. Inequality in the access to healthcare may have limited the availability of mental health resources for those in need, while gender and educational inequalities might have exacerbated existing vulnerabilities, especially among women and the youth, who have been particularly impacted by the psychosocial effects of the pandemic (Shidhaye, 2023).

## 3. Materials and Methods

The primary objective of this paper is to compile a comprehensive review of the information available to enhance our understanding of the current situation in terms of suicidal behavior. For this, a thorough search of databases and journals including PubMed, Scopus, and Web of Science was conducted. We used adapted keywords, including “suicide behavior”, “suicide in Spain”, “social stigma”, “suicide and gender”, “vulnerability”, “covid and suicide”, “sociodemographic disparities”, and others. We also carefully screened out irrelevant studies and selected relevant articles, the references of which were further scrutinized to enhance the scholarly value of this discussion. We also accessed resources and datasets from organizations such as the World Health Organization and, principally, the Spanish Statistical Office Database.

The secondary objective of this article is to explain the variations and trends seen in suicide rates in Spain during and after the COVID-19 pandemic, with a particular focus on how factors such as gender, age, and socioeconomic status influence suicidal behavior.

**Figure 1:**
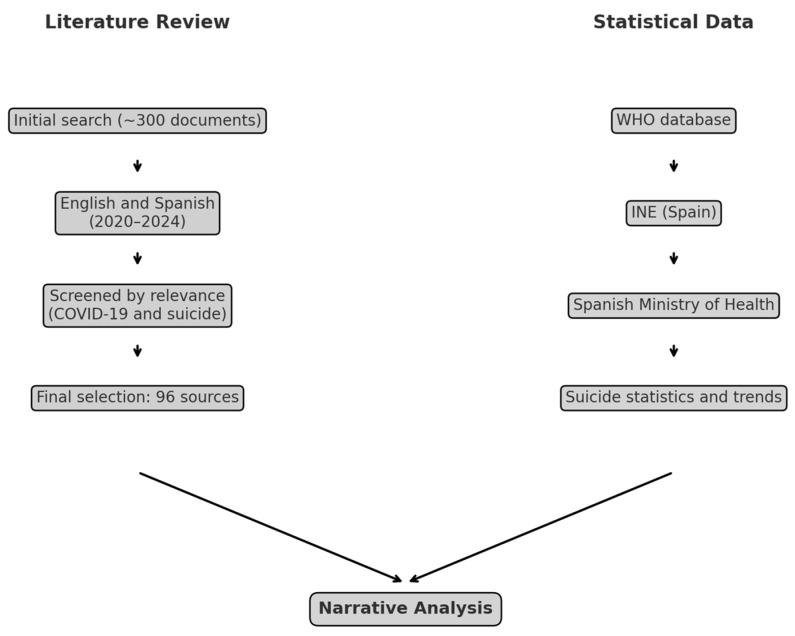
Overview of the methodological process, which included combining the NLR with statistical data from official sources. Source: self-made.

The present study is based on a narrative literature review (NLR) and official statistical data. The literature search included approximately 300 documents published between 2020 and 2024 in English and Spanish that focus on the relationship between suicide and the COVID-19 pandemic (see Figure 1). After screening for relevance, 96 sources were selected for the final analysis. In parallel, statistical data were obtained from official sources, including the World Health Organization (WHO), the Spanish National Institute of Statistics (INE), and the Ministry of Health (Spain). Both data streams were combined in an integrated narrative analysis.

This review includes studies on the incidence of suicide and suicidal behavior and utilizes a theoretical ex post facto approach to assess the effects of confinement and other restrictive measures on mental health. This study employed an NLR approach due to the broad and conceptual nature of the research objective, aiming to identify sociological patterns and contextual interpretations rather than evaluate clinical effectiveness or effect sizes. This method allows for flexible exploration of heterogeneous data sources relevant to the social determinants of suicide in the Spanish context.

## 4. Results and Analysis: Suicide in Spain During COVID-19

Suicidal behavior in Spain is a serious social and public health concern. Included in the goals proposed in the WHO Mental Health Action Plan for the period 2013–2020 was the aim to reduce suicide rates by 10% by 2020 (Mental Health and Substance Use, 2013). However, figures show that this target has not been met. In fact, in 2020, 3941 deaths by suicide were recorded, a rate of 8.4 per 100,000 members of the population, making it the leading cause of unnatural death in this region.

### 4.1. Findings from Official Data

Between 2018 and 2022, Spain recorded a sustained increase in the number of deaths by suicide within its population. During this period, the figures increased by approximately 20%, from 3539 cases in 2018 to a total of 4227 in 2022. This worrying rise reflects an alarming trend that has substantially affected the population (Instituto Nacional de Estadística, 2024). In 2023, a slight decrease in the total number of deaths by suicide was observed, as they fell to 4116. However, this overall decline was not evenly distributed across different age groups. While some segments of the population experienced a reduction, others showed an increase in cases (Ministerio de Sanidad, 2024).

Specifically, in young people aged 15–29 and adults aged 30–44 there was an increase in deaths by suicide compared to the previous year. In the 15–29 age group, there were 13 additional cases, while in the 30–44 age group, the increase was by 30 cases.

In this vein, the statistic that suicide is the leading cause of unnatural death among 15–29 year olds in Spain highlights the fact that this an emerging mental health crisis (Instituto Nacional de Estadística, 2023). This trend is a clear indicator that existing prevention strategies and support networks are insufficient to address the complexities and diverse underlying causes of death by suicide in children and youths.

The ANAR Foundation in Spain estimates that there was a 244% increase in suicide attempts and suicidal ideation and a 241% increase in self-harming behaviors among young Spaniards in 2020 (ANAR Foundation, 2021) compared to previous years, although empirical evidence has not verified these high numbers. Despite this, there has been an undeniable increase in suicidal ideation (Instituto Nacional de Estadística, 2024) and its materialization in the young population highlights the real impact that global events such as the COVID-19 pandemic have had on child and youth mental health.

In this sense, the COVID-19 crisis has significantly impacted Spanish society, not only due to its direct health consequences—such as its successive waves of infections and elevated mortality, especially during 2020—but also as a result of the restrictive public health measures implemented to contain the spread of the virus. In the field of suicide prevention, the scenario has become increasingly worrisome. Data from recent years indicate a progressive rise in deaths by suicide since 2018. Rather than interrupting this pattern, the pandemic appears to have reinforced it, with sharper increases in certain segments of the population. Specifically, 3941 individuals died by suicide in 2020, a 3.6% rise compared to 2019 and a 5.5% increase from 2018. This upward trend continued in 2021, with 4003 deaths, which was a slight increase from the previous year and still significantly above pre-pandemic levels (De la Torre Luque, 2023).

**Figure 2:**
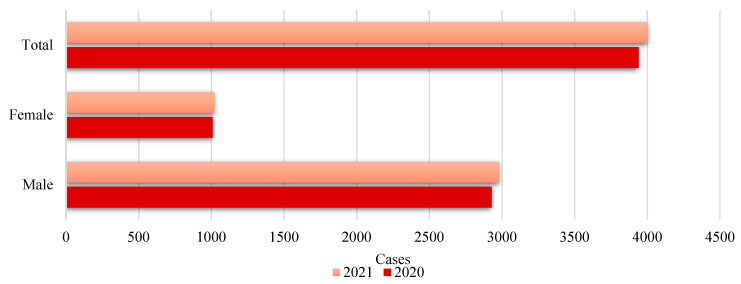
Deaths by suicide in Spain according to sex. Evolution from 2020 to 2021. Source: self-made. Instituto Nacional de Estadística (2023).

Following the height of the pandemic, an increase in the total number of deaths by suicide was observed from 2020 to 2021 (see Figure 2). This increase may be indicative of worsening mental health conditions in the general population, which could be related to the prolonged effects of the COVID-19 pandemic. These would range from socioeconomic stress to social isolation and continued uncertainty about the future.

From a gender perspective, the difference in the number of cases between men and women is substantial, with men accounting for a higher proportion of deaths by suicide. This is consistent with the sociological and biomedical literature, which indicates that while women tend to have higher rates of suicide attempts, men have higher rates of completed suicide (Freeman et al., 2017; Mościcki, 1994). The exception to this dynamic is in the youth age group (10–17 years) where, in 2021, age-adjusted mortality (standardized rate) reached 1.49 per 100,000 persons. This portion of the population presented the greatest equity in terms of gender distribution, with a proportion of 55.4% of the deaths in this group registered in males and 44.6% in females^1^.

Also, the increase in total deaths by suicide and in each of the gender categories points to 2021 being a particularly paradigmatic year. This may be linked to the late stages of the pandemic and the cumulative consequences of long periods of stress and disruption (Kim, 2022; Pathirathna et al., 2022).

These microdata also show that the highest incidence of suicide in absolute terms is found in the 45–54 age group. In 2020, this age group had one of the highest incidences of suicide in Spain, with a rate of 11.0 per 100,000 inhabitants. This population faces various socioeconomic and mental health pressures that contribute to this phenomenon. Historically, factors such as work stress, financial pressure, family responsibilities, and the onset of health problems, among other factors, are particularly relevant at this stage of life, as the literature has shown (Favril et al., 2022; Meltzer et al., 2011).

In addition, some of the studies on risk and protective factors for suicide in older people indicate that social isolation and perceived loneliness are common risk factors in older age (Ahmed & Patil, 2024; Hong et al., 2023; Motillon-Toudic et al., 2022; Solomonov et al., 2023; among others). Although this study focuses on other specific age groups, some middle-aged adults also face similar problems related to social isolation.

**Figure 3:**
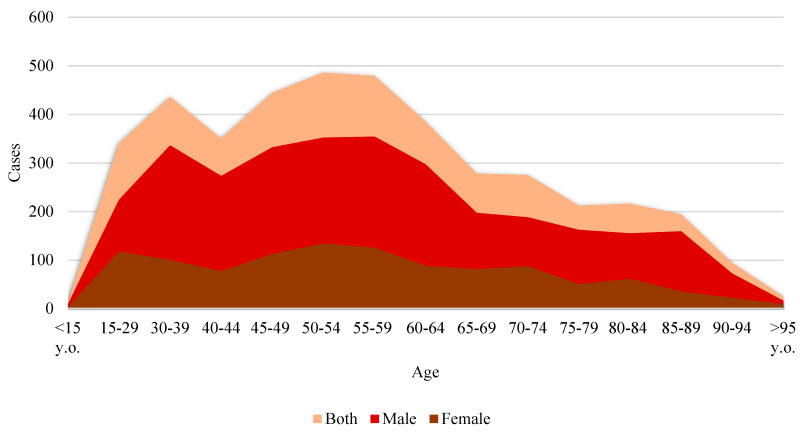
Deaths by suicide in Spain (2022) in terms of age and sex. Source: self-made. Instituto Nacional de Estadística (2023).

Younger age groups experience fewer deaths by suicide (see Figure 3), but the presence of these deaths at younger ages is still worrying for the Spanish case, indicating the need for prevention policies focused on youth mental health and the early detection of suicide risks. A gradual decline in incidence is observed with increasing age, especially after 65 years of age. This may be related to factors such as retirement, which may alleviate certain types of work and economic stress, but may also reflect a survival effect, where more resilient individuals or those with better social support survive longer.

Across all age groups in our dataset, men have higher suicide rates than women, which is consistent with trends observed globally. This is often related to cultural factors such as gender roles and the stigma associated with seeking help for mental health problems among men (Möller-Leimkühler, 2003; Schrijvers et al., 2012).

**Figure 4:**
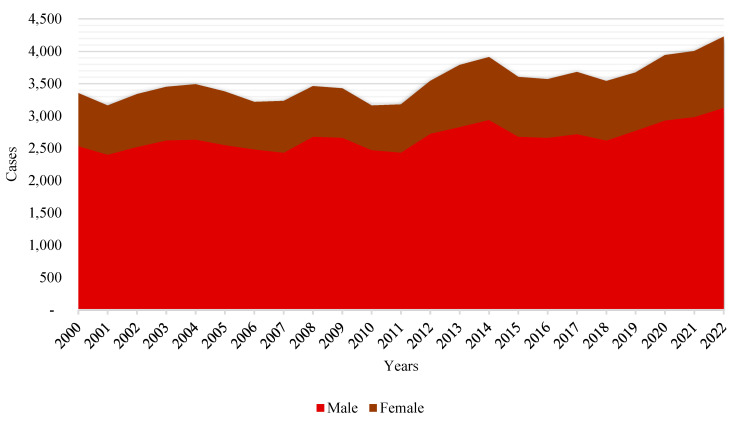
Evolution of deaths by suicide in Spain in 21st century according to sex. Source: self-made. Instituto Nacional de Estadística (2023).

Thus, in line with historic global trends, there are more deaths by suicide among men than among women recorded every year (see Figure 4). Although the numbers for both genders seem to follow a parallel trend, there are variations in the gap between the two. The gap seems to remain the same or even increase slightly over time, indicating that suicide risk factors are affecting men to a greater extent or that men are less likely to seek or receive help for mental health problems (Houle et al., 2008; Keohane & Richardson, 2018; Shi et al., 2021).

The gender gap is widest in the middle ages of life, suggesting that men in these groups may be subject to additional risk factors or have fewer mental health support networks compared to women (see Canetto & Cleary, 2012).

These trends underline the importance of considering social determinants of health and gender-specific risk factors when designing suicide prevention policies. The steady increase in cases could be indicative of deeper structural problems in society, such as unemployment, economic crises, and changes in family structure, which may contribute to social isolation and hopelessness; as the literature, particularly the sociological literature, has been warning us (Chandler, 2020; Mueller et al., 2021) from as early as Durkheim (1897/1951).

The data shown above could be related to substantial economic events, such as, for example, the 2008 financial crisis (Great Recession), which had a notable impact on Spain. The consequences of such events may include increased unemployment, economic insecurity, and job insecurity, which are known risk factors for mental health deterioration and suicide, as discussed in a multitude of papers from those years (see Chang et al., 2013; Coope et al., 2014; Corcoran et al., 2015; Harper & Bruckner, 2017; Haw et al., 2015). Using global risk theory, this increase can be interpreted as a manifestation of society’s inability to mitigate emerging psychosocial risks and provide effective safety nets. These patterns may reflect gender and age dynamics in Spanish society, where different expectations, roles, and stresses associated with each life stage and gender may contribute to mental health and suicidal behavior (see Bacigalupe et al., 2020; Toribio-Caballero et al., 2022).

### 4.2. Sociological and Theoretical Insights

The COVID-19 pandemic has demonstrated not only a capacity to affect physical health globally, but also a capacity to substantially exacerbate mental health problems, especially among those already vulnerable due to pre-existing mental disorders or poor structural conditions. In addition, the pandemic has led to a range of psychological stressors, intensifying symptoms in people with mental disorders and leading to the emergence of new clinical conditions. Social isolation, stigmatization, and information overload, a term already known as ‘infodemia’ (Caceres et al., 2022; Patwa et al., 2021; Bin Naeem & Kamel Boulos, 2021), have contributed significantly to the deterioration of mental health (Fiorillo & Gorwood, 2020). Indeed, previous research has indicated how social isolation may causally influence suicidal tendencies and how, conversely, social support may offer a protective effect, making it critical in efforts to prevent suicide (Bornheimer et al., 2020; Bränström et al., 2020).

Could this combination of factors be considered a ‘perfect storm’ for the deterioration of mental health (Reger et al., 2020)? Some of the research that has tracked the evolution of mental health since before and during the pandemic has identified increases in the frequency of depression and suicidal thoughts (Daly et al., 2022; Niedzwiedz et al., 2021; Pierce et al., 2020; Winkler et al., 2020). However, other studies have found no significant variations compared to mental health levels before the pandemic (Kwong et al., 2021). The first cohort study conducted in Spain (Ayuso-Mateos et al., 2023) found notable changes in the prevalence of depression when comparing the periods before and after the start of the first wave of the pandemic. However, no significant increase in the rates of suicidal ideation was identified in the period analyzed relative to pre-pandemic levels.

Studies focused on public health during this period have highlighted the particular vulnerability of people with mental disorders, underscoring the urgency of adapting and strengthening support systems for this population in times of crisis (Shinn & Viron, 2020). The need to investigate the interaction between COVID-19 and mental health has become imperative, particularly in terms of identifying and mitigating risk factors; improving the detection and treatment of symptoms of anxiety, depression, and stress; and assessing the impact of the pandemic on the prevalence of infections among those with and without mental disorders. This approach will not only address people’s immediate needs during health crises, but also improve the resilience and emotional well-being of people with mental disorders in the longer term.

The Spanish case is not unique in having this upward trend in recent decades (Hedegaard & Warner, 2021; Kwon et al., 2009; Martini et al., 2019; Radhakrishnan & Andrade, 2012; Tanaka & Okamoto, 2021). The analysis of deaths by suicide in this paper highlights the urgent need for suicide prevention measures. Suicide is a global public health problem affecting, to a greater or lesser extent, people of all ages, genders, and regions. Although the age-adjusted rate of deaths by suicide is currently displaying a global downward trend, not all countries are experiencing this decline. If the current trend continues, the global SDG and WHO targets to reduce deaths by suicide by one-third by 2030 will not be met.

*The Live Life: implementation guide for suicide prevention in countries* (World Health Organization, 2021) proposes four evidence-based interventions to prevent suicide. These interventions include limiting access to means of suicide, such as highly dangerous pesticides and firearms; collaborating with the media to ensure responsible coverage of suicide; promoting social–emotional skills in adolescents; and the early identification, assessment, management, and follow-up of people displaying suicidal behavior. While the WHO guidance provides a seemingly robust framework, its success depends on local adaptation and implementation, as well as continued commitment to address this problem from multiple angles. The literature has argued that some of the main problems with this approach involve addressing different social gaps (see Breet et al., 2021; Chen et al., 2024; Kasal et al., 2023; Krishnamoorthy et al., 2024; Minian et al., 2024; Werdin & Wyss, 2024).

## 5. Discussion: Suicide and the Pandemic—A Perfect Storm?

Many countries lack the resources to implement these interventions effectively. This includes funding, infrastructure, and trained personnel. It is important to increase access to mental healthcare in low-income and rural communities (World Health Organization, 2023c). The stigma associated with mental health and suicide can prevent people from seeking help. Some institutions believe that more work is needed to raise awareness and educate in order to change cultural and social perceptions (Centers for Disease Control and Prevention, 2022a). Others point out that the lack of accurate and detailed data on suicide and suicide attempts makes it difficult to plan and implement prevention strategies. Therefore, continued research is essential to better understand the risk factors involved and develop more effective interventions (Mayo Clinic, 2023). In turn, other related institutions believe that interventions must be culturally sensitive and take into account individual differences, such as age, gender, sexual orientation, and ethnicity, to be truly effective (American Psychiatric Association, 2021). One way or another, the social character of both the phenomenon and potential interventions seems to be evident. In this sense, there is a need to address the root causes of suicide, including socioeconomic conditions, trauma, substance abuse, and mental illness. Primary prevention therefore involves working on these underlying factors before suicide or suicidal behaviors manifest (National Institutes of Health, 2023). The initial identification and management of those at risk is only the first step. Continued follow-up and support is needed to ensure long-term recovery and prevent relapse (Substance Abuse and Mental Health Services Administration, 2023).

Encouraging community involvement in suicide prevention can be an effective way to reach people at risk and build support networks. This also includes general awareness-raising (Centers for Disease Control and Prevention, 2022b). Harnessing technology and digital media for suicide prevention and mental health promotion, especially among young people, is an effective strategy that has not yet been fully exploited (World Health Organization, 2023a). As shown in recent years, it is imperative to intensify, strengthen, and accelerate suicide prevention efforts to prevent further loss of life due to this public health problem (World Health Organization, 2023b). The implementation of primary and secondary prevention strategies, along with the recognition and treatment of underlying mental health conditions, is critical (Centers for Disease Control and Prevention, 2022b). This includes not only making improvements in the therapies available and early diagnosis, but also a significant increase in funding for research and prevention programs (National Institutes of Health, 2023). Adequate resource allocation is essential to develop effective and accessible interventions that can reach those at risk before they face crisis (Substance Abuse and Mental Health Services Administration, 2023).

The COVID-19 pandemic has shown that with concerted efforts and effective health planning, it is possible to address major public health challenges. However, suicide, often described as an ‘orphan disease’ due to the lack of attention and resources allocated to it, requires a similar commitment (Merayo-Cano et al., 2023). The data reinforce the need for targeted mental health interventions, especially post-pandemic, where psychological sequelae may continue to manifest. The implementation of suicide prevention and support programs should be gender-sensitive, recognizing the different needs and behaviors of men and women (Holmes et al., 2020). In addition, it has been proven necessary to examine and address the underlying causes of crisis-related increases in deaths by suicide, such as economic instability and social isolation, in order to develop more effective public policies (Pierce et al., 2020). Longitudinal studies are recommended to understand how risk trajectories change over time and to adjust interventions to make them more effective (Hawton et al., 2021).

Other lines of research have included investigating the relationship between public health interventions implemented during the pandemic and suicide rates, thus providing insights into managing future public health crises without exacerbating mental health problems (Reger et al., 2020). Prevention strategies must be designed to address the specific needs of the most vulnerable age groups, such as middle-aged adults, and must be gender-sensitive if they are to create the desired impact (Wetherall & O’Connor, 2023). It is therefore particularly important to continue researching this topic to better understand the underlying factors contributing to these trends, such as the impact of economic crises, unemployment, social isolation, and other stressors related to the pandemic and its aftermath (O’Connor et al., 2021). This will enable the development of more effective approaches to mitigating adverse effects on mental health during and after crisis situations (Kawohl & Nordt, 2020).

Although this study is based on a theoretical ex post facto analysis, it is important to acknowledge the relative scarcity of formal theoretical frameworks in the literature on the relationship between COVID-19 and suicide. Most of the available studies are descriptive or epidemiological in nature, focusing on trends and risk factors without grounding their analysis in sociological or psychological theory (Gunnell et al., 2020). This theoretical gap limits our ability to fully understand the underlying mechanisms by which large-scale social disruptions contribute to mental health crises and suicidal behavior.

In other fields, researchers have successfully used established theories to analyze the effects of COVID-19 on social outcomes. For example, Agnew’s General Strain Theory (Agnew, 1992) and Felson and Cohen’s Routine Activities Theory (Cohen & Felson, 1979) have been used to explain changes in crime rates during the pandemic. Although these theories originate in criminology, they offer a compelling illustration of how structured theoretical models can help make sense of social strain, routine disruption, and institutional breakdowns during periods of crisis. In this sense, COVID-19 should be understood not merely as a health emergency but as a disruptive social event^2^ comparable to natural disasters, wars, or economic collapses (van Bavel et al., 2020), which alter key patterns of social organization and lead to cascading consequences, including suicide, depression, and social isolation. We therefore encourage the production of more theoretically driven research to better understand how such disruptions create vulnerability, particularly among marginalized groups.

Additionally, future research would benefit from developing and testing causal models that link social disruptions like pandemics to mental health outcomes. While the current study does not propose such a model, we recognize the potential value of structural or multilevel approaches that integrate social, economic, and psychological variables. Quantitative methods, such as time series analyses, could be particularly helpful in assessing how suicide rates evolve before, during, and after crises like COVID-19. Such tools have already shown value in criminological research (Abrams, 2021) and could offer similar insights for suicide studies.

The development of these theoretical and methodological approaches would not only strengthen our understanding of the Spanish situation regarding suicides but also allow for comparisons across societies similarly affected by global health crises.

Finally, one of the main strengths of this study is its comprehensive and sociologically grounded approach, which integrates statistical data with a theoretical framework. However, its limitations include its reliance on secondary data and the absence of primary empirical data, which may constrain the depth of this causal analysis. Additionally, as a narrative review, this study is subject to selection bias in terms of the choices made regarding the inclusion of certain studies; therefore, it is necessary to continue investigating the phenomenon using quantitative sociological analyses, which continue to provide empirical evidence.

## 6. Conclusions

The study of the evolution of deaths by suicide in Spain during and after the COVID-19 pandemic reveals a series of concerning trends that demand deep reflection on the current situation and the public policies necessary to address this social and public health issue. Despite efforts to reduce suicide rates in recent years, the data reveal a continuous rise in cases, particularly among vulnerable groups such as young people and those in socioeconomically disadvantaged conditions. This trend is not isolated but rather part of a broader pattern that spans across different societies in post-pandemic contexts, highlighting the urgent need to rethink prevention approaches.

The COVID-19 pandemic has accelerated and amplified a range of pre-existing risk factors affecting mental health, such as economic instability, social isolation, and overwhelmed healthcare systems. These factors, which have disproportionately affected various segments of the population, have directly contributed to an increase in psychological disorders and suicidal behavior. In particular, social isolation, mobility restrictions, and economic uncertainty have had devastating effects on the mental health of the most vulnerable sectors, creating a breeding ground conducive to suicide. The disparity in their effects based on age, gender, and social class underscores the importance of addressing suicide not only as an individual health issue but also as a phenomenon deeply influenced by social and economic structures.

A broader cultural transformation of how societies conceptualize and address suicide and mental health disorders is required. Destigmatizing these issues is fundamental to encouraging individuals to seek help without fear of rejection or discrimination. Mental health education should be an integral part of educational and social policies, ensuring that people can identify and manage mental health risks from childhood through to adulthood. Raising awareness about suicide and supporting those with suicidal thoughts must be a priority in political, social, and educational agendas in order to create a culture of prevention that empowers individuals to take action before it is too late. From a theoretical standpoint, these findings reinforce the sociological interpretation of suicide as a phenomenon deeply rooted in structural inequalities. In conclusion, the increase in deaths by suicide in Spain during the COVID-19 pandemic highlights the urgent need for multidimensional prevention strategies that take into account structural and psychosocial factors. These findings suggest that suicide cannot be addressed solely through individual interventions, but that it must be situated within a broader sociological framework that considers economic instability, social isolation, and access to mental healthcare. These results reinforce the need to translate sociological insights into actionable policies within both public health and clinical practice.

## Data Availability

Available from the Spanish Statistical Office Database (www.ine.es).

## Notes

1 The magnitude of the gender gap remains relatively constant from one year to the next (2020–2021), suggesting that gender-associated risk factors, such as social norms about the expression of distress and access to lethal means, may not have changed significantly between these two years. For example, according to data from the US Center for Disease Control and Prevention (CDC), in 2021, the suicide rate among men was approximately four times that of women, with 22.8 deaths per 100,000 men versus 5.7 per 100,000 women. Between 2020 and 2021, an increase in suicide rates was observed for both genders. For men, the rate increased from 21.9 to 22.8 per 100,000, while for women, it rose from 5.5 to 5.7 per 100,000. This increase may be related to several factors, including the late phases of the pandemic and the cumulative consequences of long periods of stress (Centers for Disease Control and Prevention, 2024; Substance Abuse and Mental Health Services Administration, 2023).

2 Cf. The Concept of Total Social Fact, by Mauss (1925/1990), for a sociological approach.
